# 4-[(2,4-Difluoro­phen­yl)hydrazinyl­idene]-3-methyl-5-oxo-4,5-dihydro-1*H*-pyrazole-1-carbothio­amide

**DOI:** 10.1107/S1600536811044576

**Published:** 2011-10-29

**Authors:** Hoong-Kun Fun, Safra Izuani Jama Asik, Ibrahim Abdul Razak, Shobhitha Shetty, Balakrishna Kalluraya

**Affiliations:** a X-ray Crystallography Unit, School of Physics, Universiti Sains Malaysia, 11800 USM, Penang, Malaysia; b Department of Studies in Chemistry, Mangalore University, Mangalagangotri, Mangalore 574 199, India

## Abstract

In the title compound, C_11_H_9_F_2_N_5_OS, the pyrazole ring forms a dihedral angle of 16.42 (6)° with the benzene ring. Intra­molecular N—H⋯O hydrogen bonds generate two *S*(6) ring motifs. In the crystal, an *R*_2_^2^(8) ring motif is formed by a pair of inter­molecular N—H⋯S hydrogen bonds. Inter­molecular C—H⋯F hydrogen bonds further link the mol­ecules into a three-dimensional network.

## Related literature

For the biological activity of pyrazole derivatives, see: Isloor *et al.* (2009 ▶); Rai *et al.* (2008 ▶); Bradbury & Pucci (2008 ▶); Girisha *et al.* (2010 ▶). For a related structure, see: Fun *et al.* (2011 ▶). For hydrogen-bond motifs, see: Bernstein *et al.* (1995 ▶). For stability of the temperature controller used in the data collection, see: Cosier & Glazer (1986) ▶.
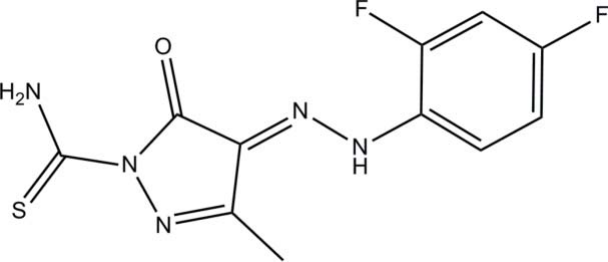


## Experimental

### 

#### Crystal data

C_11_H_9_F_2_N_5_OS
*M* *_r_* = 297.29
Triclinic, 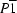

*a* = 7.9003 (1) Å
*b* = 8.2400 (1) Å
*c* = 10.1378 (1) Å
α = 103.409 (1)°
β = 99.864 (1)°
γ = 99.372 (1)°
*V* = 618.18 (1) Å^3^
*Z* = 2
Mo *K*α radiation
μ = 0.29 mm^−1^
*T* = 100 K
0.41 × 0.23 × 0.08 mm

#### Data collection

Bruker SMART APEXII CCD area-detector diffractometer
Absorption correction: multi-scan (*SADABS*; Bruker, 2009 ▶) *T*_min_ = 0.890, *T*_max_ = 0.977
15661 measured reflections
4211 independent reflections
3546 reflections with *I* > 2σ(*I*)
*R*_int_ = 0.024

#### Refinement

*R*[*F* ^2^ > 2σ(*F* ^2^)] = 0.033
*wR*(*F* ^2^) = 0.091
*S* = 1.06
4211 reflections
194 parameters
H atoms treated by a mixture of independent and constrained refinement
Δρ_max_ = 0.46 e Å^−3^
Δρ_min_ = −0.27 e Å^−3^

### 

Data collection: *APEX2* (Bruker, 2009 ▶); cell refinement: *SAINT* (Bruker, 2009 ▶); data reduction: *SAINT*; program(s) used to solve structure: *SHELXTL* (Sheldrick, 2008 ▶); program(s) used to refine structure: *SHELXTL*; molecular graphics: *SHELXTL*; software used to prepare material for publication: *SHELXTL* and *PLATON* (Spek, 2009 ▶).

## Supplementary Material

Crystal structure: contains datablock(s) global, I. DOI: 10.1107/S1600536811044576/is2796sup1.cif

Structure factors: contains datablock(s) I. DOI: 10.1107/S1600536811044576/is2796Isup2.hkl

Supplementary material file. DOI: 10.1107/S1600536811044576/is2796Isup3.cml

Additional supplementary materials:  crystallographic information; 3D view; checkCIF report

## Figures and Tables

**Table 1:** Hydrogen-bond geometry (Å, °)

*D*—H⋯*A*	*D*—H	H⋯*A*	*D*⋯*A*	*D*—H⋯*A*
N1—H1*N*1⋯O1	0.863 (17)	2.080 (17)	2.7605 (13)	135.2 (15)
N5—H1*N*5⋯S1^i^	0.842 (17)	2.607 (17)	3.4279 (11)	165.5 (15)
N5—H2*N*5⋯O1	0.880 (17)	2.048 (17)	2.7208 (13)	132.5 (14)
C10—H10*A*⋯F1^ii^	0.98	2.47	3.3016 (14)	143
C10—H10*C*⋯F1^iii^	0.98	2.53	3.2775 (14)	133
C10—H10*C*⋯F2^iv^	0.98	2.55	3.2145 (14)	125

Symmetry codes: (i) 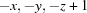; (ii) 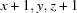; (iii) 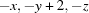; (iv) 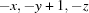.

**Fig. 1.**
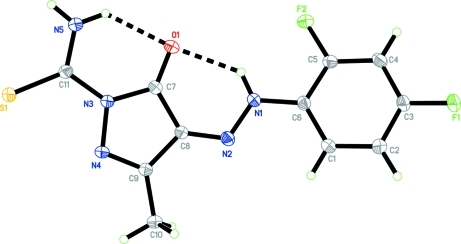
The structure of the title compound, showing 50% probability displacement ellipsoids. Hydrogen atoms are shown as spheres of arbitrary radius.

**Fig. 2.**
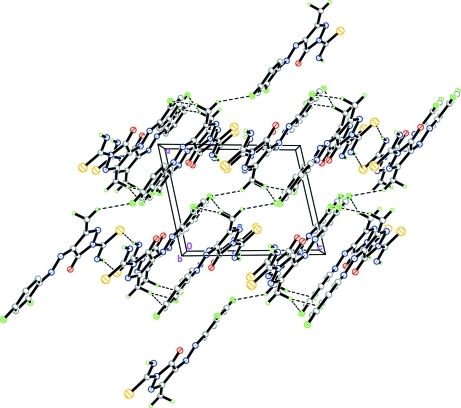
The crystal packing, viewed along the b-axis, showing the molecules formed a into three-dimensional network. Hydrogen atoms that not involved in hydrogen bonding (dashed lines) are omitted for clarity.

## supplementary crystallographic information

### Comment

The pyrazole ring is a prominent structural moiety found in numerous pharmaceutically active compounds. This is mainly due to the easy preparation and the important pharmacological activity. Therefore, the synthesis and selective functionalization of pyrazoles have been focus of active research area over the years (Isloor *et al.*, 2009). Pyrazoles have been reported to possess antibacterial activity (Rai *et al.*, 2008), and found to posses inhibitor activity against DNA gyrase and topoisomerase IV at their respective ATP-binding sites (Bradbury & Pucci, 2008). Moreover, pyrazole-containing compounds have received considerable attention owing to their diverse chemotherapeutic potentials including versatile anti-inflammatory and antimicrobial activities (Girisha *et al.*, 2010). The synthetic route followed for obtaining the title compound (I) involves the diazotization of substituted anilines  to give the diazonium salts  followed by coupling with ethyl acetoacetate in the presence of sodium acetate to give the corresponding oxobutanoate which on further reaction  with thiosemicarbazide in acetic acid gave the required thioamides.

In the title compound of (I), (Fig. 1), the pyrazole (N3/N4/C7–C9) ring is essentially planar, with a maximum deviation of 0.007 (1) Å  for atom C9 and makes a dihedral angle of 16.42 (6)° with the benzene (C1–C6) ring. The intramolecular N1—H1N1···O1 and N5—H2N5···O1 hydrogen bonds generate two S(6) ring motifs (Bernstein *et al.*, 1995). The geometric parameters are consistent to those observed in a closely related structure (Fun *et al.*, 2011).

In the crystal structure, (Fig. 2), an R_2_^2^(8) ring motif is formed by intermolecular N5—H1N5···S1 hydrogen bonds (Table 1). Intermolecular C10—H10A···F1, C10—H10C···F1 and C10—H10C···F2 (Table 1) hydrogen bonds further link the molecules into a three- dimensional network.

### Experimental

To a solution of ethyl-2-[(2,4-difluorophenyl)hydrazono]-3-oxobutanoate (0.01 mol) dissolved in glacial acetic acid (15 ml), a solution of thiosemicarbazide (0.02 mol) in glacial acetic acid (15 ml) was added and the mixture was refluxed for 4 h. It is cooled and allowed to stand overnight. The solid product that separated out was filtered and dried. It was then recrystallized from ethanol. Crystals suitable for X-ray analysis were obtained from 1:2 mixture of DMF and ethanol by slow evaporation.

### Refinement

Atoms H1N1, H1N5 and H2N5 were located in a difference Fourier map and refined freely [N—H = 0.866 (17), 0.840 (18) and 0.878 (17) Å]. The remaining H atoms were positioned geometrically (C—H = 0.95 and 0.98 Å) and refined riding on their carrier atoms. The *U*_iso_(H) values were constrained to be 1.5*U*_eq_ of the carrier atoms for methyl H atoms and 1.2*U*_eq_ for the remaining H atoms. A rotating group model was used for the methyl group.

### Crystal data

**Table:**

C_11_H_9_F_2_N_5_OS	*Z* = 2
*M**_r_* = 297.29	*F*(000) = 304
Triclinic, *P*1	*D*_x_ = 1.597 Mg m^−^^3^
Hall symbol: -P 1	Mo *K*α radiation, λ = 0.71073 Å
*a* = 7.9003 (1) Å	Cell parameters from 7383 reflections
*b* = 8.2400 (1) Å	θ = 2.6–31.9°
*c* = 10.1378 (1) Å	µ = 0.29 mm^−^^1^
α = 103.409 (1)°	*T* = 100 K
β = 99.864 (1)°	Block, green
γ = 99.372 (1)°	0.41 × 0.23 × 0.08 mm
*V* = 618.18 (1) Å^3^	

### Data collection

**Table:**

Bruker SMART APEXII CCD area-detector diffractometer	4211 independent reflections
Radiation source: fine-focus sealed tube	3546 reflections with *I* > 2σ(*I*)
graphite	*R*_int_ = 0.024
φ and ω scans	θ_max_ = 31.9°, θ_min_ = 2.1°
Absorption correction: multi-scan (*SADABS*; Bruker, 2009)	*h* = −11→11
*T*_min_ = 0.890, *T*_max_ = 0.977	*k* = −12→12
15661 measured reflections	*l* = −15→15

### Refinement

**Table:**

Refinement on *F*^2^	Primary atom site location: structure-invariant direct methods
Least-squares matrix: full	Secondary atom site location: difference Fourier map
*R*[*F*^2^ > 2σ(*F*^2^)] = 0.033	Hydrogen site location: inferred from neighbouring sites
*wR*(*F*^2^) = 0.091	H atoms treated by a mixture of independent and constrained refinement
*S* = 1.06	*w* = 1/[σ^2^(*F*_o_^2^) + (0.0469*P*)^2^ + 0.1483*P*] where *P* = (*F*_o_^2^ + 2*F*_c_^2^)/3
4211 reflections	(Δ/σ)_max_ = 0.001
194 parameters	Δρ_max_ = 0.46 e Å^−^^3^
0 restraints	Δρ_min_ = −0.27 e Å^−^^3^

### Special details

**Table:**

Experimental. The crystal was placed in the cold stream of an Oxford Cryosystems Cobra open-flow nitrogen cryostat (Cosier & Glazer, 1986) operating at 100.0 (1) K.
Geometry. All esds (except the esd in the dihedral angle between two l.s. planes) are estimated using the full covariance matrix. The cell esds are taken into account individually in the estimation of esds in distances, angles and torsion angles; correlations between esds in cell parameters are only used when they are defined by crystal symmetry. An approximate (isotropic) treatment of cell esds is used for estimating esds involving l.s. planes.
Refinement. Refinement of F^2^ against ALL reflections. The weighted R-factor wR and goodness of fit S are based on F^2^, conventional R-factors R are based on F, with F set to zero for negative F^2^. The threshold expression of F^2^ > 2sigma(F^2^) is used only for calculating R-factors(gt) etc. and is not relevant to the choice of reflections for refinement. R-factors based on F^2^ are statistically about twice as large as those based on F, and R- factors based on ALL data will be even larger.

### Fractional atomic coordinates and isotropic or equivalent isotropic displacement parameters (Å^2^)

**Table:**

	*x*	*y*	*z*	*U*_iso_*/*U*_eq_	
S1	0.21061 (4)	0.24608 (3)	0.56219 (3)	0.01893 (8)	
F1	−0.53111 (10)	0.91062 (10)	−0.26133 (8)	0.02844 (17)	
F2	−0.43017 (9)	0.41257 (8)	−0.13817 (7)	0.02208 (15)	
O1	−0.14398 (10)	0.30354 (10)	0.16468 (8)	0.01952 (16)	
N1	−0.15277 (12)	0.58736 (12)	0.06501 (10)	0.01746 (17)	
N2	−0.00803 (12)	0.66676 (12)	0.15852 (9)	0.01665 (17)	
N3	0.08671 (12)	0.38926 (11)	0.36455 (9)	0.01611 (17)	
N4	0.22017 (12)	0.53670 (11)	0.43282 (10)	0.01660 (17)	
N5	−0.06153 (13)	0.11880 (12)	0.34770 (11)	0.02077 (19)	
C1	−0.19357 (15)	0.85108 (14)	0.00178 (12)	0.0202 (2)	
H1A	−0.0949	0.9165	0.0722	0.024*	
C2	−0.29073 (16)	0.93080 (15)	−0.08133 (13)	0.0229 (2)	
H2A	−0.2603	1.0507	−0.0681	0.027*	
C3	−0.43254 (15)	0.83195 (15)	−0.18365 (12)	0.0208 (2)	
C4	−0.48169 (14)	0.65643 (14)	−0.21030 (12)	0.0190 (2)	
H4A	−0.5768	0.5907	−0.2839	0.023*	
C5	−0.38454 (14)	0.58275 (13)	−0.12371 (11)	0.01665 (19)	
C6	−0.24060 (14)	0.67551 (13)	−0.01797 (11)	0.01630 (19)	
C7	−0.01793 (13)	0.40835 (13)	0.24639 (11)	0.01574 (19)	
C8	0.05592 (13)	0.58088 (13)	0.24210 (11)	0.01541 (18)	
C9	0.20256 (14)	0.64684 (13)	0.36007 (11)	0.01583 (19)	
C10	0.32130 (15)	0.81794 (13)	0.39924 (12)	0.0200 (2)	
H10A	0.4110	0.8292	0.4825	0.030*	
H10B	0.2529	0.9064	0.4184	0.030*	
H10C	0.3783	0.8311	0.3227	0.030*	
C11	0.07145 (14)	0.24765 (13)	0.41963 (11)	0.01608 (19)	
H1N1	−0.198 (2)	0.482 (2)	0.0569 (18)	0.036 (5)*	
H1N5	−0.078 (2)	0.032 (2)	0.3780 (19)	0.036 (4)*	
H2N5	−0.138 (2)	0.126 (2)	0.2763 (18)	0.030 (4)*	

### Atomic displacement parameters (Å^2^)

**Table:**

	*U*^11^	*U*^22^	*U*^33^	*U*^12^	*U*^13^	*U*^23^
S1	0.01932 (13)	0.01622 (12)	0.01939 (14)	0.00185 (9)	−0.00104 (10)	0.00627 (10)
F1	0.0254 (4)	0.0287 (4)	0.0295 (4)	0.0030 (3)	−0.0064 (3)	0.0154 (3)
F2	0.0226 (3)	0.0156 (3)	0.0241 (3)	−0.0004 (2)	0.0008 (3)	0.0039 (3)
O1	0.0175 (4)	0.0184 (4)	0.0183 (4)	−0.0008 (3)	−0.0013 (3)	0.0032 (3)
N1	0.0172 (4)	0.0165 (4)	0.0164 (4)	0.0018 (3)	−0.0009 (3)	0.0044 (3)
N2	0.0170 (4)	0.0175 (4)	0.0143 (4)	0.0034 (3)	0.0018 (3)	0.0032 (3)
N3	0.0164 (4)	0.0135 (4)	0.0161 (4)	0.0005 (3)	−0.0002 (3)	0.0041 (3)
N4	0.0164 (4)	0.0134 (4)	0.0170 (4)	0.0000 (3)	0.0003 (3)	0.0027 (3)
N5	0.0211 (5)	0.0163 (4)	0.0216 (5)	−0.0015 (3)	−0.0015 (4)	0.0068 (4)
C1	0.0194 (5)	0.0186 (5)	0.0185 (5)	0.0011 (4)	−0.0028 (4)	0.0038 (4)
C2	0.0238 (5)	0.0183 (5)	0.0236 (6)	0.0020 (4)	−0.0022 (4)	0.0067 (4)
C3	0.0195 (5)	0.0231 (5)	0.0193 (5)	0.0038 (4)	−0.0010 (4)	0.0089 (4)
C4	0.0159 (5)	0.0220 (5)	0.0164 (5)	0.0005 (4)	0.0002 (4)	0.0046 (4)
C5	0.0166 (5)	0.0160 (4)	0.0157 (5)	0.0009 (4)	0.0030 (4)	0.0031 (4)
C6	0.0165 (4)	0.0176 (4)	0.0146 (4)	0.0036 (4)	0.0023 (4)	0.0046 (4)
C7	0.0152 (4)	0.0163 (4)	0.0147 (4)	0.0028 (3)	0.0022 (4)	0.0033 (4)
C8	0.0156 (4)	0.0146 (4)	0.0146 (4)	0.0018 (3)	0.0018 (4)	0.0030 (4)
C9	0.0164 (4)	0.0146 (4)	0.0152 (4)	0.0025 (3)	0.0022 (4)	0.0030 (4)
C10	0.0219 (5)	0.0148 (4)	0.0197 (5)	−0.0006 (4)	0.0005 (4)	0.0037 (4)
C11	0.0164 (4)	0.0143 (4)	0.0170 (5)	0.0030 (3)	0.0029 (4)	0.0041 (4)

### Geometric parameters (Å, °)

**Table:**

S1—C11	1.6633 (11)	C1—C2	1.3893 (15)
F1—C3	1.3557 (12)	C1—C6	1.3914 (15)
F2—C5	1.3564 (12)	C1—H1A	0.9500
O1—C7	1.2366 (13)	C2—C3	1.3810 (16)
N1—N2	1.3159 (13)	C2—H2A	0.9500
N1—C6	1.4016 (13)	C3—C4	1.3840 (16)
N1—H1N1	0.866 (17)	C4—C5	1.3772 (15)
N2—C8	1.3122 (13)	C4—H4A	0.9500
N3—C7	1.3905 (13)	C5—C6	1.3949 (15)
N3—C11	1.4026 (13)	C7—C8	1.4597 (14)
N3—N4	1.4176 (12)	C8—C9	1.4445 (14)
N4—C9	1.3051 (13)	C9—C10	1.4858 (14)
N5—C11	1.3334 (14)	C10—H10A	0.9800
N5—H1N5	0.840 (18)	C10—H10B	0.9800
N5—H2N5	0.878 (17)	C10—H10C	0.9800
			
N2—N1—C6	120.34 (9)	F2—C5—C6	117.21 (9)
N2—N1—H1N1	120.6 (12)	C4—C5—C6	123.13 (10)
C6—N1—H1N1	119.0 (12)	C1—C6—C5	118.51 (10)
C8—N2—N1	116.66 (9)	C1—C6—N1	123.27 (10)
C7—N3—C11	127.86 (9)	C5—C6—N1	118.20 (9)
C7—N3—N4	112.25 (8)	O1—C7—N3	127.95 (10)
C11—N3—N4	119.89 (9)	O1—C7—C8	128.37 (10)
C9—N4—N3	106.61 (8)	N3—C7—C8	103.68 (9)
C11—N5—H1N5	117.8 (12)	N2—C8—C9	126.25 (9)
C11—N5—H2N5	122.4 (11)	N2—C8—C7	127.47 (10)
H1N5—N5—H2N5	119.5 (16)	C9—C8—C7	105.88 (9)
C2—C1—C6	120.11 (10)	N4—C9—C8	111.56 (9)
C2—C1—H1A	119.9	N4—C9—C10	122.26 (10)
C6—C1—H1A	119.9	C8—C9—C10	126.18 (9)
C3—C2—C1	118.57 (10)	C9—C10—H10A	109.5
C3—C2—H2A	120.7	C9—C10—H10B	109.5
C1—C2—H2A	120.7	H10A—C10—H10B	109.5
F1—C3—C2	118.52 (10)	C9—C10—H10C	109.5
F1—C3—C4	117.86 (10)	H10A—C10—H10C	109.5
C2—C3—C4	123.62 (10)	H10B—C10—H10C	109.5
C5—C4—C3	116.00 (10)	N5—C11—N3	114.10 (9)
C5—C4—H4A	122.0	N5—C11—S1	124.50 (8)
C3—C4—H4A	122.0	N3—C11—S1	121.40 (8)
F2—C5—C4	119.65 (10)		
			
C6—N1—N2—C8	172.87 (9)	N4—N3—C7—O1	179.75 (10)
C7—N3—N4—C9	−0.44 (12)	C11—N3—C7—C8	178.66 (10)
C11—N3—N4—C9	−179.57 (9)	N4—N3—C7—C8	−0.38 (11)
C6—C1—C2—C3	−0.58 (18)	N1—N2—C8—C9	−173.72 (10)
C1—C2—C3—F1	177.63 (10)	N1—N2—C8—C7	−2.03 (16)
C1—C2—C3—C4	−1.39 (19)	O1—C7—C8—N2	7.81 (18)
F1—C3—C4—C5	−176.26 (10)	N3—C7—C8—N2	−172.05 (10)
C2—C3—C4—C5	2.77 (17)	O1—C7—C8—C9	−179.15 (10)
C3—C4—C5—F2	176.48 (10)	N3—C7—C8—C9	0.98 (11)
C3—C4—C5—C6	−2.30 (16)	N3—N4—C9—C8	1.11 (12)
C2—C1—C6—C5	1.00 (17)	N3—N4—C9—C10	−179.47 (9)
C2—C1—C6—N1	−177.62 (10)	N2—C8—C9—N4	171.79 (10)
F2—C5—C6—C1	−178.30 (10)	C7—C8—C9—N4	−1.35 (12)
C4—C5—C6—C1	0.50 (16)	N2—C8—C9—C10	−7.61 (17)
F2—C5—C6—N1	0.39 (14)	C7—C8—C9—C10	179.24 (10)
C4—C5—C6—N1	179.20 (10)	C7—N3—C11—N5	−0.44 (16)
N2—N1—C6—C1	−6.89 (16)	N4—N3—C11—N5	178.53 (9)
N2—N1—C6—C5	174.49 (9)	C7—N3—C11—S1	179.35 (8)
C11—N3—C7—O1	−1.21 (18)	N4—N3—C11—S1	−1.67 (13)

### Hydrogen-bond geometry (Å, °)

**Table:**

*D*—H···*A*	*D*—H	H···*A*	*D*···*A*	*D*—H···*A*
N1—H1N1···O1	0.863 (17)	2.080 (17)	2.7605 (13)	135.2 (15)
N5—H1N5···S1^i^	0.842 (17)	2.607 (17)	3.4279 (11)	165.5 (15)
N5—H2N5···O1	0.880 (17)	2.048 (17)	2.7208 (13)	132.5 (14)
C10—H10A···F1^ii^	0.98	2.47	3.3016 (14)	143.
C10—H10C···F1^iii^	0.98	2.53	3.2775 (14)	133.
C10—H10C···F2^iv^	0.98	2.55	3.2145 (14)	125.

Symmetry codes: (i) −*x*, −*y*, −*z*+1; (ii) *x*+1, *y*, *z*+1; (iii) −*x*, −*y*+2, −*z*; (iv) −*x*, −*y*+1, −*z*.
